# Comparison of Antimicrobial Efficacy of IKI and NaOCl Irrigants in Infected Root Canals: An In Vivo Study

**Published:** 2010-08-15

**Authors:** Abbas Abbaszadegan, Akbar Khayat, Mohammad Motamedifar

**Affiliations:** 1. Department of Endodontics, Dental School, Shiraz University of Medical Sciences, Shiraz, Iran.; 2. Department of Microbiology, Medical School, Shiraz University of Medical Sciences, Shiraz, Iran.

**Keywords:** Antibacterial, Iodine Potassium Iodide, Root Canal Irrigants, Sodium Hypochlorite

## Abstract

**INTRODUCTION:**

Effective debridement of the root canal system with chemical irrigants prior to obturation is the key to long-term success of endodontic therapy. The purpose of this study is to compare the antibacterial activity of 2.5% sodium hypochlorite (NaOCl) and 2% iodine potassium iodide (IKI) solutions as intracanal disinfectant in infected root canals during one-visit endodontic treatment procedure.

**MATERIALS AND METHODS:**

Thirty single-rooted teeth with necrotic pulps in 27 patients were selected according to specific inclusion/exclusion criteria and divided into two random groups. In group I, canals were irrigated with 2.5% NaOCl during instrumentation and in group II canals were initially irrigated with sterile saline during biomechanical preparation and then exposed to a 5-minute final irrigation with 2% IKI. Bacterial samples were taken before treatment (S_1_), and at the end of treatment (S_2_). Mann-Whitney U test was used for analysis.

**RESULTS:**

Bacteria were present in all initial samples. NaOCl was able to significantly reduce the number of colony forming units (CFU) from S_1_ to S_2_ in approximately 90% of canals. Only 15% reductions in CFUs occurred after irrigation/instrumentation in group II; this degree of disinfection was not statistically significant.

**CONCLUSION:**

According to this study, although root canal irrigation with 2.5% NaOCl could not eradicate all bacteria within the canals; it was significantly superior in comparison with 2% IKI use.

## INTRODUCTION

Bacteria and their products are regarded as the major cause of pulp and periradicular diseases [1][2]. Therefore an essential goal in endodontics is to clean, disinfect and seal the root canal system from sources of infection. The outcome of endodontic treatment largely depends on the degree of microbial control over the infections, subsequent seal and prevention of reinfection [3]. Historically, various irrigants have been used during instrumentation; the most popular has been sodium hypochlorite (NaOCl). This irrigant is a non-specific proteolytic agent with a wide range activity against endodontic microorganisms. It has excellent tissue dissolving ability and haemostatic properties [4]. Despite the favorable qualities of NaOCl, it has significant clinical disadvantages such as bad odor and taste, cytotoxicity [5][6], deteriorative effects on the mechanical and chemical properties/composition of dentine [7][8] as well as its detrimental effects on the mechanical properties and the cutting efficiency of Nickel-Titanium (NiTi) instruments [9]. It might also have negative effect on the bond strength of bonding systems [10][11]. Furthermore, it can bring severe tissue reaction following inadvertent injection beyond the apex [12].

Iodine potassium iodide (IKI) root canal disinfectant when used as 2% solution, has acceptable antimicrobial properties, more pleasant odor, taste and lower toxicity when compared with NaOCl [6][13][14][15]. It can also penetrate deep, up to 1000 µm into dentin when irrigated for 5 minutes [15].

Sirén et al. used bovine root blocks infected with Enterococcus (E) faecalis to examine the effectiveness of 2% IKI. Their results showed no bacterial growth up to 700 μm and 950 μm at one and 7 day incubations [16].

Safavi et al. demonstrated the efficacy of IKI irrigant in eliminating E. faecalis when used for 10 minute in infected dentinal tubules following NaOCl irrigation [17]. Calcium hydroxide (Ca(OH)_2_) required 24 hours to be as effective. A recent similar investigation by Lin et al. indicated that IKI is a better chemical agent for disinfection than calcium hydroxide in shorter exposure times [18].

A clinical study on root-filled teeth with apical periodontitis (failed endodontic cases) demonstrated that a 5 minute final flush with IKI following 2.5% NaOCl irrigation completely eradicated bacterial in all but one case [19].

In one randomized clinical trial, the anti microbial efficacy of endodontic procedures performed in one-visit was compared with two-visit treatments. In single visit treatment they exposed canals to 5% IKI for 10 minutes while they used Ca(OH)_2_ medicament in the two-visit group. Results showed that residual microorganisms were recovered in 29% and 36% of the treated teeth with one-visit and two-visit procedures respectively [20].

Despite these early promising results there are insufficient clinical studies to recommend IKI as a suitable irrigant alternative. This study aimed to compare the antibacterial activity of NaOCl with 2% IKI solution in infected root canals following one-visit endodontic procedure.

## MATERIALS AND METHODS

Approval for the study protocol was obtained from the Ethics Committee of Shiraz University of Medical Sciences. Patients presenting to the endodontic clinic at Shiraz School of Dentistry with a single rooted tooth, for evaluation and treatment of apical periodontitis were considered for this study.

Thirty single rooted teeth from 27 patients (9 maxillary central incisors, 7 maxillary lateral incisors, 4 second maxillary premolars, 2 mandibular incisors, 1 mandibular canine, and 7 mandibular premolars), that is 7 males and 20 females, aged 20–64 years old were selected for this study. The inclusion criteria were teeth with single roots and necrotic pulps as confirmed by negative response to sensitivity pulp tests. Patients who received antibiotic therapy within the past 3 months were excluded as well as teeth with extensive caries or fractures of the root/crown, teeth with a history of previous endodontic treatment, and cases showing periodontal pockets over 4 mm depth. Possible complications/risks were explained to patients and informed consents obtained before starting the treatment.

Before isolation with rubber dam, teeth with supragingival plaque were scaled and cleaned with pumice. Caries/coronal restorations were then removed with sterile burs. Rubber dam isolation was carried out and the operative fields were cleaned with 30% hydrogen peroxide followed by a 2.5% NaOCl swab. After access cavity preparation, the operative fields were again disinfected as described. Then 5% sodium thiosulphate was used to deactivate any remnant NaOCl [21].

Cases were randomly divided in to 2 groups (n=15). The first root canal sampling (S_1_) was carried out with sterile paper points #15 which were placed in the canal about 1 mm short of the radiographic apex to absorb the fluid in the canal. Each paper point remained in the canal for at least 1 minute. Paper points were then transferred to tubes containing 2 mL of reduced transport fluid (thioglycollate broth). Working length was measured with electronic apex locator (VDW, Munich, Germany) and all canals were prepared with Protaper rotary files (Dentsply Maillefer Tulsa, Okla) up to size F3.

***Group I:***In this group irrigation was performed with 2 mL 2.5% NaOCl after each instrument (10mL overall). Irrigant was delivered in the canals by means of a 5mL disposable syringe with a 27-gauge needle. Apical patency was also confirmed with a small file (K-file #10 or #15) throughout the procedures after each larger file size. After instrumentation was completed, the canals were flushed with 2 mL 5% sodium thiosulfate followed by 2 mL normal saline and second microbial sampling (S_2_) was achieved by using 3 sterile #30 paper cones.

***Group II:***During instrumentation irrigation was carried out with normal saline. Two milliliters of this solution were used to irrigate the canals after each instrument (10mL overall). Apical patency was checked during instrumentation. Each canal was flushed with 2% IKI solution as final rinse for a period of 5 minutes. After inactivation of IKI with 2mL 5% sodium thiosulfate followed by 2mL normal saline, the second microbial sampling (S_2_) was performed.

Subsequently, the canals were filled with gutta-percha and Tubli-Seal sealer (ZOE-based; SybronEndo, Orange, CA, USA) using cold lateral compaction technique. The tooth was temporized and permanent restoration was planned at the next visit. All clinical procedures were conducted by one person.

Samples were transported to the laboratory within maximum of 1 hour for microbiological processing. Samples in reduced transport fluid vials were dispersed within a vortex for 30 seconds and 10-fold serial dilutions up to 103 and 102 were made in pre-reduced anaerobic sterilized buffered salt solution. Aliquots of 100 µL from the undiluted suspension and the highest dilution were each spread on Brucella agar plates (Merck, Germany) which were enriched with defibrinated sheep blood (5%), hemin (5 mg/L) and menadione (1 mg/L). Plates were then incubated anaerobically within anaerobic jars (GasPak system, Anaerocult A, Merck, Germany) at 37°C for 7 days. After incubation, the total colony-forming units (CFUs) were counted in each 1/100 and 1/1000 dilutions, and mean bacterial colony calculated based on the known dilution factors.

### Statistical Analysis

Usefulness of each treatment in reducing cultivable bacteria was accounted as percentage of CFU reduction based on quantitative data obtained from samples S_1_ and S_2_. Quantitative data were statistically analyzed for differences by using the Mann-Whitney U test (P<0.05).

## RESULTS

One case in group II (case 5) was excluded because of inaccuracies during laboratorial procedures. Bacteria were detected in initial samples of both experimental groups. The median value of the number of CFU in the initial samples was 1.48×10^5^ per mL, ranging from 1.7×10^4^ to 1.19×10^6^.

As summarized in Table 1, after chemo-mechanical preparation using NaOCl, 2 of the 15 canals (13.3%) showed negative culture results. When compared with initial samples (S_1_), chemomechanical preparation reduced the number of bacteria by approximately 90%.

**Table 1:** Bacterial load (CFU per µL) and percent reduction in two groups.

****		**S_1_**	**S_2_**	**%R** **(S_1_-S_2_)**	**P value**
**Group I (NaOCl)**	1	40	4	90	
	2	79.5	17	78.6	
	3	73.5	16.5	77.5	
	4	1150	6.5	99.4	
	5	375	84.5	77.4	
	6	1075	6.5	99.3	
	7	195	11.5	94.1	
	8	145	35	75.8	
	9	235	7.5	96.8	
	10	715	13.5	98.1	
	11	1190	22.5	98.1	
	12	212.5	13.5	93.6	
	13	55	0	100	
	14	189	32.5	82.8	
	15	148.5	0	100	
	**Mean**			90.8	0.001
**Group II (IKI)**	1	36.5	97	-165.7	
	2	20	26	-30	
	3	80	13.5	83.1	
	4	24	14.5	39.5	
	5	-	-	-	
	6	130	231.5	-78.0	
	7	17	6	64.7	
	8	51.5	4	92.2	
	9	28.5	17	40.3	
	10	155	45	70.9	
	11	187	137.5	26.4	
	12	113	19	83.1	
	13	192	174	9.3	
	14	205	282.5	-37.8	
	15	161.5	142.5	11.7	
	**Mean**			15.0	0.245

In group II, only 15% CFU reduction compared to initial sample was achieved after instrumentation and irrigation. There were no bacteria free cultures in S_2_.

**Figure 1:**
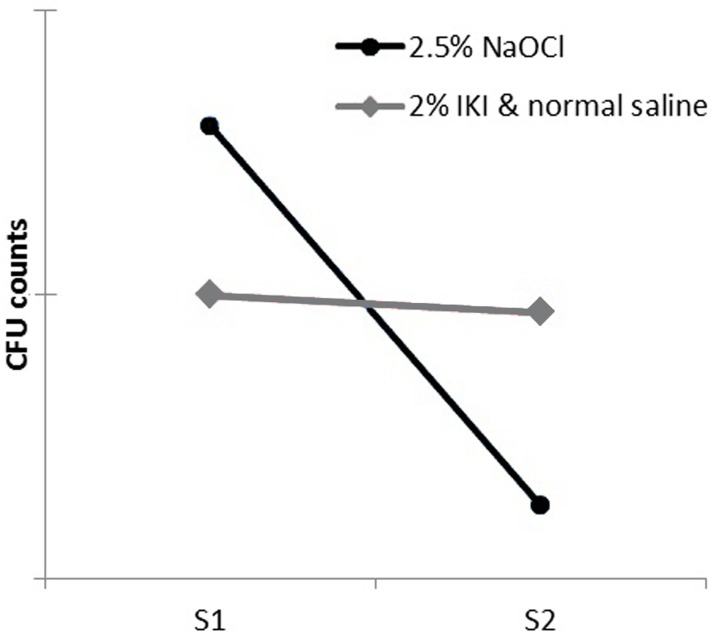
Comparison of CFU reduction after using NaOCl and IKI following normal saline.

Statistical analysis revealed that the CFU count reduction from S1 to S2 was significant for group I while it was not significant for group II (Figure 1).

## DISCUSSION

Successful treatment of endodontic infections is strongly dependent on the eradication of microbial infection from the root canal system. Therefore, the absence or presence of bacteria in the root canals prior to obturation is useful information that can help evaluate treatment efficacy.

Culturing technique was used in the present investigation to compare the antimicrobial effectiveness of NaOCl with 2% IKI solution final rinse following normal saline irrigation.

Culturing procedure is one of the most reliable methods to detect viable bacteria after antimicrobial treatment. Furthermore, studies have shown a positive relation between negative cultures and optimal treatment outcome [3][22].

However, an important shortcoming of the method refers to the fact that about half of the endodontic-related bacteria have not been yet cultivated by culturing techniques [23]. Moreover, culturing does not show the exact bacterial virulence factors, which might be more important than the quantity of bacteria growth in apical periodontitis.

In this study, all root canals harbored bacteria before treatment, confirming the strong correlation between bacteria and apical pathology. Figdor and Sundqvist revealed that infected root canal systems can harbor <10^2^ to >10^8^ per mL bacterial cells; similar amounts were also found in this study, i.e.10^4^ to 10^6^ per mL bacterial cells (mean 2.5×10^5^) [24].

The best known and most commonly used irrigant is NaOCl. Clinical efficacy of this irrigant comes from its ability to dissolve necrotic and organic tissues and its antimicrobial properties [13].

Siqueira et al. showed that chemomechanical preparation with 2.5% NaOCl significantly reduced the number of bacteria but could not render the canals bacteria free in more than one-half of the cases [25].

More recent clinical trial revealed that 1.3% NaOCl is capable of decreasing bacterial load up to 95%; however, approximately 30% to 40% of teeth had remnant bacteria in post chemo-mechanical samples [26].

Our results are somewhat consistent with previous findings and demonstrate that 2.5% NaOCl can on average reduce intracanal bacteria by 90%. In 86.7% of cases bacteria was not completely eradicated by NaOCl; only two cases (13.3%) did not show bacteria.

A negative culture however, does not necessarily indicate sterility [27]. On the contrary, a negative culture usually means that cultivable bacterial populations have dropped to levels which are not detectable by current culture dependent methods and these levels can be compatible to periradicular healing in most cases [28].

In this study, group II involved initial irrigation with saline during instrumentation in order to determine the efficacy of IKI solely. Also this helped to prevent possible staining of dentin due to long exposures to IKI. In this group the mean CFU reduction from S1 to S2 was as low as 15%. This might be related to the fact that normal saline and IKI have little or no effect on organic tissues like necrotic pulp whereas NaOCl has more than 90% effectiveness in dissolving organic tissues [29]. It is possible that some parts of remnant necrotic pulp tissues containing bacteria have been transferred to transport media by paper points.

Bacterial species in a biofilm nature can be 100 to 1000 fold more resistant to antimicrobial agents when compared to planktonic nature [30]. It seems that NaOCl is the most effective endodontic irrigant in microbial biofilm disruption [4]. NaOCl dissolves organic components of smear layer and exposes inorganic material [31][32] which can lead to biofilm removal.

Sayin et al. demonstrated that 2% IKI had much less effect on removing Ca^2+^ from root dentin in comparison with different concentrations of NaOCl in different time intervals [33]. Therefore, the inferior outcomes of IKI can be attributed to its ineffectiveness on bacteria in biofilm nature in the infected root canal. In addition, a limited 5 minute contact time of IKI as final rinse might be insufficient to kill the microorganisms in the extensive biofilms of necrotic pulps. Hence, using this solution for an extended time or as an irrigant from the beginning of the treatment, with the risk of possible staining, may reveal different results.

The previous reported success of IKI may be due to the fact that initial irrigation was conducted with NaOCl, which might have enhanced its bacterial effectiveness.

A form of bacterial regrowth after receiving a 5 minute final rinse of saline and MTAD following 1.3% NaOCl canal irrigation has been reported by Malkhassian et al. [26]. The authors failed to present reasonable explanation for this impaired effect other than the limited number of samples, prior exposure of dentin to NaOCl and detergent constituent of MTAD that may support the growth of specific bacteria.

One hypothesis is that short-term final rinses have residual effectiveness beyond their application; that is, they may have the ability to detach bacteria from root canal walls but not enough time to destroy them before sampling procedures. Moreover, a larger sample size is needed to achieve adequate statistical power for more precise findings.

Though the initial CFU count of canals which were irrigated with NaOCl had decreased about by 90%; viable microorganisms were still detectable by culturing in post instrumentation samples.

It is not known whether remnant bacteria area in the range of body immune system compatibility.

Furthermore, it seems that current instruments and irrigation are not enough to remove all microorganisms in unreachable areas of the root canal system in a single visit. While it seems that remnant microorganisms would not be able to cause harm when they became entombed by the obturation material; there is little evidence for this assumption [34].

## CONCLUSION

Although in vitro models are useful to evaluate the potency of antimicrobial agents and their spectrum of activity, testing them under in vivo conditions may show different results that challenge our expectation.

In the present in vivo study, 2.5% NaOCl irrigation could not completely eradicate bacteria but was significantly superior to normal saline followed by 2% IKI final rinse.
